# The Effects of a Low-Carbohydrate Diet *vs.* a Low-Fat Diet on Novel Cardiovascular Risk Factors: A Randomized Controlled Trial

**DOI:** 10.3390/nu7095377

**Published:** 2015-09-17

**Authors:** Tian Hu, Lu Yao, Kristi Reynolds, Paul K. Whelton, Tianhua Niu, Shengxu Li, Jiang He, Lydia A. Bazzano

**Affiliations:** 1 Department of Epidemiology, School of Public Health and Tropical Medicine, Tulane University 1440 Canal Street, Suite 2000, New Orleans, LA 70112, USA; E-Mails: pkwhelton@gmail.com (P.K.W.); sli10@tulane.edu (S.L.); jhe@tulane.edu (J.H.); lbazzano@tulane.edu (L.A.B.); 2 Division of Epidemiology and Community Health, University of Minnesota, 1300 S 2nd Street, Suite 300, Minneapolis, MN 55454, USA; E-Mail: yaoxx097@umn.edu; 3 Department of Research & Evaluation, Kaiser Permanente Southern California, 100 South Los Robles, 2nd Floor, Pasadena, CA 91101, USA; E-Mail: Kristi.Reynolds@kp.org; 4 Department of Biostatistics and Bioinformatics, School of Public Health and Tropical Medicine, Tulane University 1440 Canal Street, Suite 2001, New Orleans, LA 70112, USA; E-Mail: tniu@tulane.edu

**Keywords:** dietary carbohydrate, clinical trial, nutrition, obesity, inflammation, endothelial dysfunction, adipocytokines

## Abstract

Increasing evidence supports a low-carbohydrate diet for weight loss and improvement in traditional cardiovascular disease (CVD) markers. Effects on novel CVD markers remain unclear. We examined the effects of a low-carbohydrate diet (<40 g/day; *n* = 75) *versus* a low-fat diet (<30% kcal/day from total fat, <7% saturated fat; *n* = 73) on biomarkers representing inflammation, adipocyte dysfunction, and endothelial dysfunction in a 12 month clinical trial among 148 obese adults free of diabetes and CVD. Participants met with a study dietitian on a periodic basis and each diet group received the same behavioral curriculum which included dietary instruction and supportive counseling. Eighty percent of participants completed the intervention. At 12 months, participants on the low-carbohydrate diet had significantly greater increases in adiponectin (mean difference in change, 1336 ng/mL (95% CI, 342 to 2330 ng/mL); *p* = 0.009) and greater decreases in intercellular adhesion molecule-1 concentrations (−16.8 ng/mL (−32.0 to −1.6 ng/mL); *p* = 0.031) than those on the low-fat diet. Changes in other novel CVD markers were not significantly different between groups. In conclusion, despite the differences in weight changes on diets, a low-carbohydrate diet resulted in similar or greater improvement in inflammation, adipocyte dysfunction, and endothelial dysfunction than a standard low-fat diet among obese persons.

## 1. Introduction

Cardiovascular disease (CVD) is a leading cause of death and a major cause of disability in the United States [1]. A large body of evidence has suggested that inflammation and endothelial dysfunction are the initial detectable steps in the process of developing CVD [2,3,4,5,6]. Many novel biochemical markers associated with CVD, such as adipocytokines and cellular adhesion molecules, have been implicated in the processes of systemic inflammation, intracellular signaling and cell migration and, as such, may serve to promote the development of atherosclerotic lesions [3]. Adipocytokines are secreted from adipose cells, and regulate metabolic pathways throughout the body [2,7,8]. Some adipocytokines, such as adiponectin and leptin, also play a role in vascular inflammation [2,3,9]. Since endothelial dysfunction results in increased permeability of the vascular system to leukocytes and other cellular components, soluble circulating forms of cellular adhesion molecules reflect local endothelial activation [5,10,11,12,13,14]. Assessing these novel biomarkers allows for the early detection of changes which may ultimately affect CVD risk and outcomes.

Primary prevention strategies for CVD focus on lifestyle modification, including dietary interventions. Increasing evidence suggests that a low-carbohydrate diet may result in both weight loss and improvement in traditional CVD risk factors such as blood pressure, lipids, glucose, and insulin [15,16,17,18]. Results from clinical trials of typical low-carbohydrate diets have demonstrated that, compared to currently-recommended low-fat diets, low-carbohydrate diets produced greater weight loss and more favorable changes in body composition, high-density lipoprotein cholesterol, and triglycerides [15,19], while similar or lesser reductions in low-density lipoprotein cholesterol, blood pressure, blood glucose or insulin [15,17,20,21,22,23]. The favorable changes in these CVD risk factors induced by low-carbohydrate diets are even independent of weight loss [24]. However, the effects of a low-carbohydrate diet on novel CVD risk factors remain unclear due to sparse and inconsistent data from studies typically limited by small sample sizes [19,25], relatively short durations [26,27,28,29], and low completion or high dropout rates [30,31,32]. Hence, we examined the effects of a 12-month low-carbohydrate as compared to a low-fat dietary intervention on adipocytokines, and biochemical markers of inflammation and endothelial biomarkers.

## 2. Experimental Section

The study (Trial Registration: Clinicaltrials.gov; Identifier: NCT00609271) design and methods are presented in detail elsewhere and described briefly here [17]. All procedures were approved by the Institutional Review Board of Tulane University Health Sciences Center and all participants provided written informed consent.

### 2.1. Setting and Participants

In brief, the trial was designed to examine the effects of a low-carbohydrate diet on body weight and cardiovascular risk factors. Men and women 22–75 years of age with a body mass index of 30 to 45 kg/m^2^ who lived in the Greater New Orleans Area were recruited using mailing lists, flyers, work site and community screenings, and television advertisements. Individuals who had type 2 diabetes, CVD or chronic renal disease at baseline were excluded, as were those who were currently using prescription weight-loss medications, undergoing weight loss surgery, or had experienced significant weight loss within six months of study entry. Participants were recruited, enrolled, and followed from 2008 to 2011 at the Tulane University Health Sciences Center in New Orleans, Louisiana.

### 2.2. Intervention

Participants with obesity were randomly assigned to either a low-carbohydrate diet where net carbohydrate intake (total carbohydrate minus total fiber) was restricted to<40 grams/day, or a low-fat diet which restricted total fat to <30% of daily energy, with <7% from saturated fat (consistent with national guidelines) [33,34]. Participants met with a dietitian in weekly individual counseling sessions for the first month, followed by small group counseling sessions every other week for the subsequent five months, and then monthly for the last six months. Individual sessions lasted 1 h and included supportive counseling and dietary instructions in the form of recipes. Group sessions were held separately for participants in the low-fat and low-carbohydrate groups but participants received the same dietary behavioral curriculum, which included identical information on dietary fiber intake and education on the different types of fat with an emphasis on the benefits of monounsaturated fats and recommendations to limit or eliminate trans-fats. Behavioral counseling also emphasized portion control and change in eating patterns. An optional daily low-carbohydrate or low-fat meal replacement (bar or shake) was provided to participants in each group for the duration of the intervention. Participants were counseled to maintain their baseline levels of physical activity, which was assessed using validated measures at each clinical visit.

### 2.3. Data Collection

Blood samples were collected after the participant had fasted for 12 h. Novel CVD risk factors were measured at baseline and at three, six, and 12 months of intervention. The study endpoints included inflammatory biomarkers (interleukin (IL)-6, IL-8, tumor necrosis factor (TNF)-α, adipocytokines (adiponectin, leptin, resistin), and biochemical markers of endothelial function (Intercellular Adhesion Molecule (ICAM)-1, vascular cell adhesion molecule (VCAM)-1 and E-selectin). Plasma IL-6, IL-8, and TNF-α were measured using a multiplex assay (Milliplex Adipokine Human Panel B, HADK2-61K-B03, EDM Millipore, Billerica, MA, USA). The inter-assay coefficients of variation ranged from 6.97% to 10.37% for IL-6, 5.12% to 11.81% for IL-8, and 3.89% to 9.89% for TNF-α, respectively. Plasma adiponectin, leptin, and resistin were measured by ELISA (Catalog#: SRP300 for adiponectin, SLP00 for leptin, and SRSN00 for resistin, R & D Systems, Minneapolis, MN, USA). The inter-assay coefficients of variation ranged from 6.23% to 13.70% for adiponectin, 2.13% to 4.27% for leptin, and 5.16% to 11.19% for resistin, respectively. Plasma ICAM-1, VCAM-1, and E-resistin were measured by ELISA (Catalog#: SCD540 for ICAM-1, SVC00 for VCAM-1, and SSLE00 for E-selectin, R & D Systems, Minneapolis, MN, USA). The inter-assay coefficients of variation ranged from 2.54% to 6.82% for ICAM-1, 3.11% to 4.68% for VCAM-1, and 4.52% to 12.59% for E-selectin, respectively.

As previously described, a detailed medical history including medication use, health behaviors, and physical activity was obtained at baseline [17]. Two 24 h dietary recalls, one on a week day and the other on a weekend day, were obtained from each participant by a trained and certified study dietitian at baseline and at each follow-up examination. Dietary nutrient intakes were calculated using the food composition tables included in the Nutrition Data System for Research [35]. For the purposes of quality control, five percent of dietary recalls were recorded and reviewed. Urinary ketones were measured by dipstick at each dietary counseling session and each follow-up visit to assess adherence to a low-carbohydrate diet [36].

### 2.4. Statistical Analysis

The population characteristics and novel CVD risk factors at baseline were expressed as mean (SD) or N (%) by intervention group. We used Student’s *t*-tests, Wilcoxon-Mann-Whitney tests, or Chi-square tests to compare baseline characteristics between the two groups. Data on dietary composition were expressed as means (SDs) and compared using *t*-tests at baseline and each follow-up visit. To examine change in each novel CVD risk factor across four time points over 12 months, we used a mixed-effects model that included an indicator variable for time (3, 6, and 12 months), diet group, an interaction term for diet group by time, and baseline value of the corresponding endpoint. This modeling technique allows missing data under the assumption of missing-at-random. We performed sensitivity analysis to assess the robustness of our conclusions and departures from the missing-at-random assumption. We used multiple imputation techniques to impute missing values including additional covariates (age, sex, race, marital status, education, and employment status) in the model to make the missing-at-random assumption more plausible [37]. To account for the effects of weight loss on changes in novel CVD markers, we performed a mediation analysis to assess the proportion of dietary effects that is explained by differences in weight loss between the two groups. We applied the conventional mediation analysis method since there was no interaction between weight loss, the potential mediator, and diet group in the mixed-effect models (all *p*-values > 0.05) [38,39]. This conventional approach first estimated the residual effect between diet group and outcome concentrations after adjustment for weight loss and then determined the mediating effect by subtracting this from the total effect [40]. All analyses were conducted using the intention-to-treat principle. All *p*-values were two-sided and statistical significance was defined as *p* < 0.05. We used SAS (version 9.3; SAS Institute Inc, Cary, NC, USA) for all analyses.

## 3. Results

We randomized 148 obese adults (mean age 46.8 years, 11.5% men, and 51% African-Americans) to the two diet groups. At 3, 6, and 12 months, 93.2%, 87.7%, and 82.2% of participants in the low-fat group and 92.0%, 82.7%, and 78.7% of participants in the low-carbohydrate group, respectively, completed assessments. Characteristics of the trial participants are shown in Table 1. Demographic, behavioral, traditional and novel CVD risk factors were comparable between the low-carbohydrate and low-fat diet groups prior to commencement of the intervention. On average, participants in the low-carbohydrate group lost 5.3 kg and those in the low-fat group lost 1.5 kg at 12 months (Table A1) 

**Table 1:** Baseline characteristics of trial participants.

Variable ^*^	LFD (*n* = 73)	LCD (*n* = 75)
Age, year	47.8 (10.4)	45.8 (9.9)
Female	65 (89)	66 (88)
Race/Ethnicity		
White	33 (45)	34 (45)
African-American	36 (49)	40 (53)
Asian	0 (0)	1 (1)
Hispanic	3 (4)	0 (0)
Others	1 (1)	0 (0)
Weight, kg	97.9 (13.5)	96.3 (12.7)
Waist circumference, cm	111.0 (10.7)	108.4 (9.3)
Systolic blood pressure, mmHg	124.9 (13.8)	120.3 (12.8)
Diastolic blood pressure, mmHg	79.4 (8.3)	77.5 (9.0)
LDL cholesterol, mg/dL	122.7 (38.6)	122.5 (34.6)
HDL cholesterol, mg/dL	56.5 (12.8)	53.8 (13.3)
Plasma glucose, mg/dL	93.4 (9.2)	94.5 (10.9)
Anti-hypertensive medication use	24 (32.9)	21 (28.0)
Lipid-lowering medication use	9 (12.3)	12 (16.0)
Physical activity, MET-hour/week ^†^	19.6 (35.5)	16.3 (26.0)
C-reactive protein, mg/L	4.9 (5.1)	4.9 (4.2)
IL-6, pg/mL	3.6 (3.0)	3.4 (2.4)
IL-8, pg/mL	2.3 (1.4)	2.1 (1.3)
TNF-α, pg/mL	2.6 (1.9)	2.2 (1.4)
Adiponectin, ng/mL	7692 (4425)	8203 (5442)
Leptin, ng/mL	51.6 (26)	45.4 (23.2)
Resistin, ng/mL	9.1 (4.7)	8.2 (3.6)
ICAM-1, ng/mL	215 (101)	221 (73)
VCAM-1, ng/mL	628 (154)	642 (186)
E-selectin, ng/mL	45.2 (18.9)	47.1 (21.7)

HDL: high-density lipoprotein; ICAM-1: soluble intercellular adhesion molecule-1; IL: interleukin; LCD: low-carbohydrate diet; LFD: low-fat diet; LDL: low-density lipoprotein; MET: metabolic equivalent; TNF: tumor necrosis factor; VCAM-1: soluble vascular adhesion molecule-1; ^*^: All data were expressed as N (%), mean (standard deviation), or median (interquartile range) when appropriate; ^†^: Physical activity was calculated as the sum of hours per week of moderate to vigorous activities (walking, sports, dance and conditioning) multiplied by the activity’s individual MET value.

### 3.1. Dietary Measurements

At baseline, 3, 6, and 12 months, 139, 123, 104, and 103 participants provided 24 h dietary recalls. Data on daily dietary composition for participants is presented in Table 2. There were no significant differences in total energy or macronutrient intake between the low-carbohydrate and low-fat groups at baseline. At 12 months, the intake of carbohydrate was significantly lower and intakes of protein or fats (total, saturated, and unsaturated fats) were significantly higher in the low-carbohydrate group, compared to the low-fat group (*p* < 0.001 for these comparisons). Further details of dietary measurements have been published previously [17].

### 3.2. Changes in Novel Cardiovascular Risk Factors

Predicted mean differences (95% CIs) in changes in inflammatory biomarkers, adipocytokines, and endothelial biomarkers from baseline are shown by assigned dietary group in Table 3. Changes in IL-6, IL-8, or TNF- α between the two groups were not statistically significant at 12 months.

Throughout the study, adiponectin concentrations increased significantly in both the low-fat and low-carbohydrate groups, and the increases in the low-carbohydrate group were significantly greater than that observed in the low-fat group (mean difference in change at 12 months, 1336 ng/mL (95% CI, 342 to 2330 ng/mL); *p* = 0.009). Decreases in leptin were greater in the low-carbohydrate group at three and six months, but did not differ significantly from those in the low-fat group at 12 months. Changes in resistin did not differ significantly between the two groups.

ICAM-1 increased significantly at each clinical assessment in the low-fat group but not in the low-carbohydrate group. The mean difference in change at 12 months was −16.8 ng/mL (95% CI, −32.0 to −1.6 ng/mL; *p* = 0.031). Changes in VCAM-1 did not differ significantly between the two groups. Decreases in E-selectin were greater in the low-carbohydrate group at three and six months than in the low-fat diet, but the changes did not differ significantly between the groups at 12 months.

As previously reported the proportion of participants with detectable urinary ketone levels was significantly higher in the low-carbohydrate group than in the low-fat group and no significant differences in physical activity were identified between groups throughout the study [17].

Mediation analysis showed that 53.6% of the difference in adiponectin and 69.5% of the difference in ICAM-1 were not explained by differences in weight loss between the two diet groups at 12 months. We examined differences among Caucasian and African-American participants and found similar trends between the two race groups (Table A2 and Table A3). Results of sensitivity analyses using multiple imputation techniques to impute missing values were consistent with those presented in our primary analyses.

**Table 2:** Daily dietary composition in the low-fat and low-carbohydrate diet groups over the course of the study ^*^.

Nutrients	Baseline		3 Month		6 Month		12 Month	
	LFD (*n* = 69)	LCD (*n* = 70)	LFD (*n* = 61)	LCD (*n* = 62)	LFD (*n* = 50)	LCD (*n* = 54)	LFD (*n* = 49)	LCD (*n* = 54)
Energy, kcal	2034 (702)	1998 (740)	1418 (468)	1258 (409) ^†^	1481 (483)	1324 (537)	1527 (522)	1448 (610)
Carbohydrate								
Carb, g	242 (100)	242 (92)	193 (75)	97 (45) ^†^	202 (79)	93 (46) ^†^	198 (78)	127 (69) ^†^
Carb, % kcal	46.0 (7.8)	48.1 (8.8)	52.9 (10.7)	28.9 (12.6) ^†^	52.4 (8.9)	27.5 (12.1) ^†^	54.0 (9.6)	34.0 (13.9)
Total fiber, g	16.7 (6.6)	18.5 (8.7)	16.9 (8.9)	16.2 (8.9)	16.4 (8.1)	15.1 (7.5)	15.6 (7.7)	15.1 (8.7)
Fat								
Total fat, g	80.7 (32.4)	75.6 (36.4)	45.3 (21.7)	62.6 (28.6) ^†^	46.4 (18.9)	67.2 (38.2) ^†^	52.4 (24.3)	69.0 (36.8) ^†^
SFA, g	27.6 (13.6)	24.7 (14.4)	13.5 (6.8)	19.9 (9.8) ^†^	14.0 (6.7)	21.2 (15.6) ^†^	15.8 (8.4)	23.3 (15.1) ^†^
MUFA, g	29.3 (12.5)	28.1 (13.7)	17.0 (9.5)	24.0 (12.0) ^†^	17.1 (7.3)	25.1 (13.6) ^†^	20.0 (9.9)	25.8 (14.3) ^†^
PUFA, g	17.1 (8.1)	16.7 (9.2)	10.9 (6.2)	13.3 (6.9) ^†^	11.3 (5.5)	15.0 (8.8) ^†^	12.2 (7.7)	14.2 (7.4) ^†^
Total fat, % kcal	34.7 (6.6)	32.5 (7.2)	27.5 (8.8)	42.7 (10.0) ^†^	27.9 (7.3)	43.4 (11.8) ^†^	29.8 (8.8)	40.7 (10.6) ^†^
SFA, % kcal	11.6 (2.9)	10.5 (3.4) ^†^	8.1 (3.0)	13.6 (4.2) ^†^	8.4 (2.9)	13.4 (4.5) ^†^	9.0 (3.2)	13.4 (4.8) ^†^
MUFA, % kcal	12.7 (3.0)	12.0 (3.1)	10.3 (4.2)	16.3 (4.4) ^†^	10.3 (3.1)	16.3 (4.9) ^†^	11.3 (3.7)	15.3 (4.7) ^†^
PUFA, % kcal	7.5 (2.7)	7.3 (2.7)	6.7 (3.1)	9.1 (3.1) ^†^	6.7 (2.2)	9.8 (4.3) ^†^	6.9 (3.5)	(3.3) ^†^
Protein								
Protein, % kcal	17.6 (5.2)	17.3 (5.0)	19.0 (5.7)	25.6 (7.7) ^†^	18.3 (5.0)	26.3 (5.6) ^†^	18.6 (5.8)	23.6 (7.4) ^†^
Folate, mg	0.40 (0.17)	0.41 (0.19)	0.36 (0.23)	0.29 (0.13)	0.38 (0.22)	0.32 (0.17)	0.35 (0.20)	0.31 (0.15)
β-carotene, mg ^‡^	0.75 (2.04)	0.49 (1.31)	0.99 (2.03)	0.89 (1.99)	0.74 (2.44)	0.60 (1.50)	1.00 (2.35)	0.42 (1.42)
Vitamin C, mg	78.4 (47.0)	88.6 (60.0)	82.7 (68.1)	67.8 (45.2)	85.4 (66.0)	81.1 (54.8)	82.5 (61.1)	72.5 (67.9)

LCD: low-carbohydrate diet; LFD: low-fat diet; MUFA: monounsaturated fatty acid; PUFA: polyunsaturated fatty acid; SFA: saturated fatty acid; ^*^: Data were expressed as mean (standard deviation) unless otherwise indicated; ^†^: *p* < 0.05, for the difference between the two groups at time point; ^‡^: β-carotene was expressed as median (interquartile range).

**Table 3:** Predicted mean difference in changes in novel cardiovascular biomarkers from baseline, by assigned diet group.

	Predicted Mean Difference (95% CI) ^*^			*p*-Value ^†^
	LFD (*n* = 73)	LCD (*n* = 75)	Mean Difference in Change	
Inflammatory Biomarkers				
IL-6, pg/mL				
3 month	0.84 (0.30, 1.38)	0.65 (0.13, 1.18)	−0.19 (−0.94, 0.56)	0.63
6 month	1.16 (0.55, 1.76)	0.63 (0.03, 1.22)	−0.53 (−1.41, 0.32)	0.22
12 month	1.80 (0.87, 2.72)	0.58 (−0.33, 1.49)	−1.22 (−2.52, 0.08)	0.066
IL-8, pg/mL				
3 month	−0.04 (−0.25, 0.17)	−0.10 (−0.30, 0.10)	−0.06 (−0.35, 0.23)	0.69
6 month	−0.03 (−0.19, 0.13)	−0.14 (−0.30, 0.01)	−0.11 (−0.34, 0.12)	0.33
12 month	−0.02 (−0.19, 0.15)	−0.23 (−0.40, −0.06)	−0.22 (−0.45, 0.02)	0.078
TNF-α, pg/mL				
3 month	1.09 (0.50, 1.68)	0.38 (−0.20, 0.96)	−0.71 (−1.54, 0.12)	0.093
6 month	1.09 (0.66, 1.52)	0.50 (0.08, 0.92)	−0.59 (−1.19, 0.02)	0.057
12 month	1.08 (0.70, 1.47)	0.74 (0.36, 1.13)	−0.34 (−0.89, 0.21)	0.22
Adipocytokines				
Adiponectin, ng/mL				
3 month	934 (413, 1456)	1563 (1051, 2076)	629 (−102, 1360)	0.091
6 month	1025 (553, 1498)	1890 (1423, 2357)	865 (200, 1529)	0.011
12 month	1208 (503, 1912)	2544 (1843, 3245)	1336 (342, 2330)	0.009
Leptin, ng/mL				
3 month	−3.5 (−7.3, 0.3)	−14.7 (−18.4, −11.0)	−11.2 (−16.5, −5.9)	<0.001
6 month	−2.6 (−6.1, 0.8)	−11.6 (−15.0, −8.2)	−9.0 (−13.9, −4.1)	<0.001
12 month	−1.0 (−5.5, 3.6)	−5.4 (−9.9, −1.0)	−4.5 (−10.9, 1.9)	0.166
Resistin, ng/mL				
3 month	1.68 (1.18, 2.19)	1.24 (0.74, 1.74)	−0.45 (−1.16, 0.26)	0.22
6 month	1.27 (0.87, 1.67)	0.84 (0.44, 1.24)	−0.43 (−1.00, 0.14)	0.137
12 month	0.44 (0.04, 0.84)	0.04 (−0.36, 0.44)	−0.40 (−0.97, 0.17)	0.171
Endothelial Biomarkers				
ICAM-1, ng/mL				
3 month	13.5 (1.1, 25.9)	6.2 (−6.0, 18.5)	−7.3 (−24.7, 10.1)	0.41
6 month	15.0 (3.8, 26.3)	4.6 (−6.6, 15.8)	−10.4 (−26.3, 5.4)	0.194
12 month	18.1 (7.3, 28.8)	1.3 (−9.5, 12.0)	−16.8 (−32.0, −1.6)	0.031
VCAM-1, ng/mL				
3 month	40.4 (14.6, 66.3)	35.3 (5.1, 60.5)	−5.1 (−41.2, 31.0)	0.78
6 month	19.6 (−3.1, 42.3)	19.5 (−2.8, 41.7)	−0.1 (−31.9, 31.7)	0.99
12 month	−22.2 (−51.4, 7.1)	−12.2 (−41.3, 17.0)	10.0 (−31.4, 51.3)	0.63
E-selectin, ng/mL				
3 month	−3.2 (−5.1, −1.2)	−7.7 (−9.6, −5.8)	−4.5 (−7.3, −1.8)	0.002
6 month	−0.7 (−2.8, 1.4)	−5.0 (−7.1, −2.9)	−4.3 (−7.2, −1.4)	0.005
12 month	4.2 (0.5, 7.9)	0.4 (−3.3, 4.1)	−3.8 (−9.1, 1.4)	0.146

ICAM: intercellular adhesion molecule; IL: interleukin; LCD: low-carbohydrate diet; LFD: low-fat diet; TNF: tumor necrosis factor; VCAM: vascular adhesion molecule. ^*^: From the random effects models (including diet and time) and expressed as mean (95% confidence interval); **^†^**: *p*-values for the difference between the two groups at each time point.

## 4. Discussion

This randomized controlled trial suggests that a 12 month low-carbohydrate diet results in more favorable changes than a low-fat diet in adiponectin and ICAM-1 concentrations, and does not differ from a low-fat diet in reducing other adipocytokines or biochemical markers of endothelial dysfunction in an obese adult population. The two diets had equivalent effects on IL-6, IL-8, and TNF-α concentrations. These findings as a whole suggest that a low-carbohydrate diet is equivalent to, or more effective than, a low-fat diet for improving some novel CVD risk factors. Notably, mediation analysis indicated that approximately 60%–70% of dietary effects on novel CVD risk factors were not explained by differences in weight loss and therefore were plausibly due to different macronutrient concentrations in the diet. This finding is important, because it indicates that obese adults who lose weight on a low-carbohydrate diet can improve inflammatory status, endothelial function, and adipocyte function, to the same or greater degree than those on a low-fat diet. The significance of this study is manifested by identifying changes in inflammatory biomarkers, adipocytokines, and biochemical markers of endothelial dysfunction on low-carbohydrate and low-fat diets, thereby investigating the mechanism of their dietary effects on CVD. This study also has important public health implications in the setting of a high prevalence of excessive refined carbohydrate consumption and an epidemic of obesity and CVD worldwide.

To our knowledge, our study is the largest randomized controlled trial to comprehensively examine the effect of a low-carbohydrate diet as compared to a low-fat diet for weight loss on novel CVD risk factors throughout the intervention period. Our study is also unique in having a racially diverse population with 51% African American participants. Examining novel CVD risk factors comprehensively may provide further information on mechanisms through which weight loss with these different diets potentially reduces CVD risk. A few small and short-term trials reported a similar pattern for most but not all adipocytokines, inflammatory markers, and markers for endothelial function [19,25,26,27,28,29,31,32]. For example, a three month intervention study reported that a very low-carbohydrate diet (12% of energy from carbohydrates daily) resulted in more reduction in IL-6, IL-8, and TNF-alpha concentrations than did in a low-fat diet (25% fat, with 10% saturated fat) in 40 young adults with dyslipidemias [29]. Another study showed equivalent changes in adiponectin between the two diets; however, that study had a small sample size (*n* < 60) and low completion rate (approximately 40%) [32]. Significant differences in changes in ICAM-1 between the low-carbohydrate and low-fat diets were not observed in a study where participants were followed for only 1.5 months [28]. In contrast with previous studies, our study included 148 non-diabetic obese participants with a substantial proportion of African-Americans (a group under-represented in previous trials) and had a high completion rate (approximately 80%) over 12 months of follow-up.

While both low-carbohydrate and low-fat diets can lead to weight loss and have a beneficial effect on CVD risk profile, there may be different mechanisms by which the diets and weight loss on these diets potentially reduces CVD risk. Carbohydrate consumption leading to insulin secretion may create a pro-inflammatory milieu, particularly in the setting of obesity where insulin resistance is common. A low-carbohydrate diet may improve adipocyte function due to the increase in insulin sensitivity with weight loss as well as decreases in insulin secretion due to dietary consumption of carbohydrates [41]. Adiponectin has both insulin sensitizing and anti-inflammatory properties [42]. Some studies have shown that adiponectin concentrations increase when weight loss is induced by low-carbohydrate diets but not low-fat, high carbohydrate diets [27,43,44]. Low-carbohydrate diets may reduce fat mass to a greater degree than low-fat diets [17], and reductions in fat mass are associated with increases in adiponectin concentrations [45,46]. Low-carbohydrate diets may also elevate gene expression of adiponectin [47]. Likewise, increase in insulin sensitivity may contribute to the improvement in endothelial function on a low-carbohydrate diet [48]. Like our study, a previous study also demonstrated significant decreases ICAM-1, with no significant difference in VCAM-1 or E-selectin on the two diets [32]. The reason for these differing effects is not clear, but suggests these biochemical markers of endothelial dysfunction may be regulated by alternate independent mechanisms. The greater increases in HDL concentrations on a low-carbohydrate diet, as compared with a low-fat diet [18], may contribute to the differences in changes in ICAM-1 concentrations between the two diets. It has been reported that individuals with higher HDL concentrations had significantly lower concentrations of soluble ICAM-1 [49]. In addition, high intake of dietary fructose may stimulate endothelial inflammatory processes by up-regulating ICAM-1 through increase in ICAM-1 mRNA and protein expression [50]. In contrast, no evidence has suggested a link between dietary carbohydrates and VCAM-1 or E-selectin.

The strengths of our study included analysis of a large, diverse population of obese participants with comprehensive, 12 month follow-up and examinations which included assessments of novel CVD risk factors at each time point, blinded outcome assessment, high completion rate, high rates of dietary adherence by 24 h recalls and urinary ketone levels. Our conclusions are subject to some limitations. This clinical trial was not powered to detect changes in these novel CVD risk factors. With 148 participants, however, there was sufficient statistical power to detect the effects of the dietary intervention on the novel CVD risk factors. The clinical significance of small changes in these novel CVD risk factors is unknown. Self-reported dietary information may be subject to recall bias; however, we collected this information within 24 h of dietary intake and used multiple 24 h dietary recalls to reflect eating patterns on weekdays and weekends at each time point. Dietitians were not blinded to the study hypothesis; however, specific and detailed scripts were used in all counseling sessions, and dietary sessions for both groups were intermittently observed for consistency by an independent registered dietitian consultant who was not a regular part of the study staff. Statistically significant results should be interpreted with caution due to the number of comparisons tested in the analyses, particularly for *p*-values denoting significance levels close to 0.05. The sample with a large proportion of female participants may limit generalizability of the results.

In conclusion, findings from this year-long clinical trial indicate that, despite the differences in weight changes on a low-carbohydrate diet and a low-fat diet, a low-carbohydrate diet resulted in similar or greater improvement in inflammation, endothelial dysfunction and adipocyte dysfunction than a standard low-fat diet for obese adults. Identification of such patterns and mechanisms may provide the basis for successful personalized and public health nutrition recommendations for CVD prevention. Further studies should continue to evaluate cardiovascular effects of a low-carbohydrate diet and explore the potential mechanisms.

## Appendix

**Table A1:** Predicted mean change in body weight by assigned dietary group ^*^.

Predicted Mean Change	3-month Change, kg	6-month Change, kg	12-month Change, kg
Low-carbohydrate diet (*N* = 75)	−5.7 (−6.5, −4.9)	−5.6 (−6.5, −4.6)	−5.3 (−6.8, −3.8)
Low-fat diet (*N* = 73)	−2.6 (−3.4, −1.7)	−2.3 (−3.3, −1.3)	−1.8 (−3.3, −0.3)
Mean difference in changes	−3.1 (−4.3, −1.9)	−3.2 (−4.6, −1.9)	−3.5 (−5.6, −1.4)
*p* value ^†^	<0.001	<0.001	0.002

^*^ The predicted mean difference in weight change is from the mixed-effects models (including diet, time and diet group by time interaction term) and expressed as mean (95% confidence interval). ^†^ *p* values for the difference between the two groups at each time point.

**Table A2:** Predicted mean difference in changes in novel cardiovascular markers from baseline by assigned dietary group among Caucasians **^*^**.

Novel Cardiovascular Markers	Predicted Mean Difference (95% CI) ^*^			
	Low-Fat Diet (*N* = 73)	Low-Carbohydrate Diet (*N* = 75)	Mean Difference in Change	*p* Value ^†^
Inflammatory biomarkers				
IL-6, pg/mL				
3 month	−0.49 (−3.10, 2.11)	−0.73 (−3.35, 1.89)	−0.23 (−3.94, 3.47)	0.90
6 month	0.45 (−1.68, 2.58)	−1.10 (−3.24, 1.04)	−1.54 (−4.57, 1.48)	0.31
12 month	2.33 (−1.13, 5.79)	−1.84 (−5.31, 1.63)	−4.17 (−9.07, 0.73)	0.093
IL-8, pg/mL				
3 month	−1.31 (−2.40, −0.22)	−0.49 (−1.57, 0.59)	0.82 (−0.71, 2.35)	0.29
6 month	0.72 (−1.06, 2.50)	−0.57 (−2.35, 1.21)	−1.29 (−3.81, 1.23)	0.31
12 month	4.8 (−2.64, 12.20)	−0.73 (−8.11, 6.66)	−5.51 (−15.97, 4.96)	0.29
TNF-α, pg/mL				
3 month	0.54 (0.03, 1.06)	0.49 (−0.02, 1.00)	−0.05 (−0.78, 0.68)	0.89
6 month	0.81 (0.35, 1.27)	0.53 (0.08, 0.99)	−0.27 (−0.92, 0.38)	0.40
12 month	1.35 (0.74,1.95)	0.62 (0.02, 1.23)	−0.72 (−1.58, 0.13)	0.096
Adipocytokines				
Adiponectin, ng/mL				
3 month	1082 (309, 1856)	1544 (778, 2310)	462 (−627, 1550)	0.40
6 month	1270 (478, 2063)	2086 (1299, 2873)	816 (−301, 1932)	0.148
12 month	1647 (338, 2956)	3171 (1868, 4474)	1524 (−323, 3371)	0.103
Leptin, ng/mL				
3 month	−5.0 (−9.2, −0.8)	−15.7 (−19.9, −11.5)	−10.7 (−16.7, −4.7)	0.001
6 month	−4.4 (−8.2, −0.6)	−13.3 (−17.1, −9.5)	−8.9 (−14.3, −3.6)	0.002
12 month	−3.1 (−9.2, 3.1)	−8.5 (−14.7, −2.3)	−5.4 (−14.2, 3.4)	0.22
Resistin, ng/mL				
3 month	1.61 (1.03, 2.19)	1.14 (0.56, 1.72)	−0.47 (−1.29, 0.35)	0.25
6 month	1.28 (0.81, 1.76)	0.80 (0.33, 1.27)	−0.48 (−1.15, 0.19)	0.152
12 month	0.63 (−0.01, 1.27)	0.12 (−0.52, 0.76)	−0.51 (−1.42, 0.39)	0.26
Endothelial biomarkers				
ICAM-1, ng/mL				
3 month	10.6 (−10.3, 31.5)	5.8 (−14.8, 26.4)	−4.8 (−34.1, 24.6)	0.74
6 month	10.5 (−7.9, 28.9)	3.7 (−14.5, 21.8)	−6.8 (−32.7, 19.1)	0.60
12 month	10.2 (−6.8, 27.3)	−0.7 (−17.6, 16.3)	−10.9 (−34.9, 13.1)	0.37
VCAM-1, ng/mL				
3 month	70.6 (20.2, 120.9)	54.0 (3.9, 104.1)	−16.6 (−87.6, 54.5)	0.64
6 month	51.2 (5.4, 96.9)	30.4 (−15.2, 76.0)	−20.8 (−85.5, 43.9)	0.52
12 month	12.4 (−48.4, 73.2)	−16.8 (−78.6, 44.9)	−29.2 (−116.0, 57.6)	0.50
E-selectin, ng/mL				
3 month	−3.1 (−6.4, 0.1)	−8.9 (−12.2, −5.6)	−5.7 (−10.4, −1.1)	0.016
6 month	−1.7 (−5.6, 2.1)	−6.6 (−10.4, −2.8)	−4.9 (−10.3, 0.5)	0.076
12 month	1.1 (−5.3, 7.5)	−2.1 (−8.5, 4.3)	−3.2 (−12.2, 5.8)	0.48

ICAM: intercellular adhesion molecule; VCAM: vascular adhesion molecule. **^*^** The predicted mean difference in changes in endothelial biomarkers are from the random effects models (including diet and time) and expressed as mean (95% confidence interval). **^†^** *p* values for the difference between the two groups at each time point.

**Table A3:** Predicted mean difference in changes in novel cardiovascular markers from baseline by assigned dietary group among African-Americans **^*^**.

Novel Cardiovascular Markers	Predicted Mean Difference (95% CI) ^*^			
	Low-Fat Diet (*N* = 73)	Low-Carbohydrate Diet (*N* = 75)	Mean Difference in Change	*p* Value ^†^
Inflammatory biomarkers				
IL-6, pg/mL				
3 month	1.53 (0.68, 2.37)	0.88 (0.07, 1.68)	−0.65 (−1.82, 0.53)	0.27
6 month	1.34 (0.14, 2.53)	0.81 (−0.35, 1.98)	−0.52 (−2.20, 1.15)	0.53
12 month	0.96 (−1.35, 3.27)	0.68 (−1.59, 2.95)	−0.28 (−3.52, 2.96)	0.86
IL-8, pg/mL				
3 month	0.09 (−0.22, 0.40)	−0.13 (−0.42, 0.16)	−0.22 (−0.65, 0.21)	0.31
6 month	0.04 (−0.21, 0.28)	−0.16 (−0.40, 0.07)	−0.20 (−0.54, 0.14)	0.25
12 month	−0.07 (−0.36, 0.22)	−0.23 (−0.51, 0.05)	−0.16 (−0.56, 0.25)	0.44
TNF-α, pg/mL				
3 month	1.72 (0.60, 2.83)	0.21 (−0.84, 1.25)	−1.51 (−3.04, 0.02)	0.053
6 month	1.42 (0.66, 2.19)	0.41 (−0.31, 1.13)	−1.01 (−2.06, 0.04)	0.059
12 month	0.83 (0.34, 1.32)	0.82 (0.34, 1.29)	−0.01 (−0.70, 0.67)	0.97
Adipocytokines				
Adiponectin, ng/mL				
3 month	884 (131, 1638)	1605 (903, 2308)	721 (−309, 1751)	0.167
6 month	951 (304, 1598)	1765 (1154, 2376)	814 (−77, 1704)	0.073
12 month	1085 (167, 2003)	2084 (1196, 2972)	999 (−278, 2277)	0.123
Leptin, ng/mL				
3 month	−1.3 (−7.9, 5.3)	−14.2 (−20.3, −8.1)	−12.9 (−22.0, −3.8)	0.006
6 month	−0.3 (−6.3, 5.7)	−10.6 (−16.2, −5.0)	−10.3 (−18.6, −2.0)	0.016
12 month	1.7 (−5.3, 8.7)	−3.4 (−10.1, 3.3)	−5.1 (−14.9, 4.7)	0.30
Resistin, ng/mL				
3 month	1.72 (0.85, 2.59)	1.31 (0.49, 2.14)	−0.41 (−1.61, 0.80)	0.50
6 month	1.26 (0.55, 1.97)	0.89 (0.22, 1.56)	−0.37 (−1.35, 0.61)	0.46
12 month	0.34 (−0.28, 0.95)	0.04 (−0.54, 0.63)	−0.30 (−1.15, 0.56)	0.49
Endothelial biomarkers				
ICAM-1, ng/mL				
3 month	15.4 (−1.2, 32.1)	5.3 (−10.5, 21.2)	−10.1 (−33.1, 13.0)	0.38
6 month	17.5 (1.3, 33.7)	5.3 (−10.2, 20.7)	−12.2 (−34.7, 10.3)	0.28
12 month	21.6 (4.8, 38.5)	5.1 (−11.0, 21.3)	−16.5 (−39.9, 6.9)	0.164
VCAM-1, ng/mL				
3 month	23.7 (−1.8, 49.2)	10.3 (−13.5, 34.1)	−13.4 (−48.1, 21.5)	0.45
6 month	3.4 (−21.5, 28.4)	4.2 (−19.5, 27.9)	0.8 (−33.6, 35.2)	0.96
12 month	−37.1 (−72.5, −1.6)	−7.9 (−42.2, 26.3)	29.1 (−20.2, 78.4)	0.24
E-selectin, ng/mL				
3 month	−3.1 (−5.7, −0.4)	−6.9 (−9.4, −4.5)	−3.9 (−7.4, −0.3)	0.035
6 month	0.4 (−2.1, 2.9)	−3.7 (−6.1, −1.4)	−4.1 (−7.5, −0.7)	0.018
12 month	7.4 (2.7, 12.0)	2.7 (−1.8, 7.2)	−4.7 (−11.2, 1.8)	0.152

ICAM: intercellular adhesion molecule; VCAM: vascular adhesion molecule. **^*^** The predicted mean changes in endothelial biomarkers are from the random effects models (including diet and time) and expressed as mean (95% confidence interval). **^†^** *p* values for the difference between the two groups at each time point.

## References

[1] Mozaffarian D., Benjamin E.J., Go A.S., Arnett D.K., Blaha M.J., Cushman M., de Ferranti S., Despres J.P., Fullerton H.J., Howard V.J. (2015). Heart disease and stroke statistics—2015 update: A report from the American Heart Association. Circulation.

[2] Balagopal P.B., de Ferranti S.D., Cook S., Daniels S.R., Gidding S.S., Hayman L.L., McCrindle B.W., Mietus-Snyder M.L., Steinberger J. (2011). Nontraditional risk factors and biomarkers for cardiovascular disease: Mechanistic, research, and clinical considerations for youth: A scientific statement from the American Heart Association. Circulation.

[3] Pearson T.A., Mensah G.A., Alexander R.W., Anderson J.L., Cannon R.O., Criqui M., Fadl Y.Y., Fortmann S.P., Hong Y., Myers G.L. (2003). Markers of inflammation and cardiovascular disease: Application to clinical and public health practice: A statement for healthcare professionals from the centers for disease control and prevention and the American Heart Association. Circulation.

[4] Wang T.J. (2008). New cardiovascular risk factors exist, but are they clinically useful?. Eur. Heart J..

[5] Falk E. (2006). Pathogenesis of atherosclerosis. J. Am. Coll. Cardiol..

[6] Libby P., Okamoto Y., Rocha V.Z., Folco E. (2010). Inflammation in atherosclerosis: Transition from theory to practice. Circ. J..

[7] Ng T.W., Watts G.F., Farvid M.S., Chan D.C., Barrett P.H. (2005). Adipocytokines and VLDL metabolism: Independent regulatory effects of adiponectin, insulin resistance, and fat compartments on VLDL apolipoprotein B-100 kinetics?. Diabetes.

[8] Kanaya A.M., Fyr C.W., Vittinghoff E., Harris T.B., Park S.W., Goodpaster B.H., Tylavsky F., Cummings S.R. (2006). Adipocytokines and incident diabetes mellitus in older adults: The independent effect of plasminogen activator inhibitor 1. Arch. Intern. Med..

[9] Mohamed-Ali V., Pinkney J.H., Coppack S.W. (1998). Adipose tissue as an endocrine and paracrine organ. Int. J. Obes. Relat. Metab. Disord..

[10] Bevilacqua M.P., Nelson R.M. (1993). Selectins. J. Clin. Investig..

[11] Jang Y., Lincoff A.M., Plow E.F., Topol E.J. (1994). Cell adhesion molecules in coronary artery disease. J. Am. Coll. Cardiol..

[12] Springer T.A. (1990). Adhesion receptors of the immune system. Nature.

[13] Gearing A.J., Hemingway I., Pigott R., Hughes J., Rees A.J., Cashman S.J. (1992). Soluble forms of vascular adhesion molecules, E-selectin, ICAM-1, and VCAM-1: Pathological significance. Ann. N. Y. Acad. Sci..

[14] Ballantyne C.M. (2002). Soluble adhesion molecules and coronary heart disease. Lancet.

[15] Gardner C.D., Kiazand A., Alhassan S., Kim S., Stafford R.S., Balise R.R., Kraemer H.C., King A.C. (2007). Comparison of the Atkins, Zone, Ornish, and LEARN diets for change in weight and related risk factors among overweight premenopausal women: The A to Z weight loss study: A randomized trial. J. Am. Med. Assoc..

[16] Atkins R.C. (2002). Dr. Atkins’ New Diet Revolution.

[17] Bazzano L.A., Hu T., Reynolds K., Yao L., Bunol C., Liu Y., Chen C.S., Klag M.J., Whelton P.K., He J. (2014). Effects of low-carbohydrate and low-fat diets: A randomized trial. Ann. Intern. Med..

[18] Hu T., Mills K.T., Yao L., Demanelis K., Eloustaz M., Yancy W.S., Kelly T.N., He J., Bazzano L.A. (2012). Effects of low-carbohydrate diets versus low-fat diets on metabolic risk factors: A meta-analysis of randomized controlled clinical trials. Am. J. Epidemiol..

[19] Brehm B.J., Seeley R.J., Daniels S.R., D’Alessio D.A. (2003). A randomized trial comparing a very low carbohydrate diet and a calorie-restricted low fat diet on body weight and cardiovascular risk factors in healthy women. J. Clin. Endocrinol. Metab..

[20] Brinkworth G.D., Noakes M., Buckley J.D., Keogh J.B., Clifton P.M. (2009). Long-term effects of a very-low-carbohydrate weight loss diet compared with an isocaloric low-fat diet after 12 mo. Am. J. Clin. Nutr..

[21] Foster G.D., Wyatt H.R., Hill J.O., Makris A.P., Rosenbaum D.L., Brill C., Stein R.I., Mohammed B.S., Miller B., Rader D.J. (2010). Weight and metabolic outcomes after 2 years on a low-carbohydrate versus low-fat diet: A randomized trial. Ann. Intern. Med..

[22] Yancy W.S., Olsen M.K., Guyton J.R., Bakst R.P., Westman E.C. (2004). A low-carbohydrate, ketogenic diet versus a low-fat diet to treat obesity and hyperlipidemia: A randomized, controlled trial. Ann. Intern. Med..

[23] Shai I., Schwarzfuchs D., Henkin Y., Shahar D.R., Witkow S., Greenberg I., Golan R., Fraser D., Bolotin A., Vardi H. (2008). Weight loss with a low-carbohydrate, mediterranean, or low-fat diet. N. Engl. J. Med..

[24] Feinman R.D., Pogozelski W.K., Astrup A., Bernstein R.K., Fine E.J., Westman E.C., Accurso A., Frassetto L., Gower B.A., McFarlane S.I. (2015). Dietary carbohydrate restriction as the first approach in diabetes management: Critical review and evidence base. Nutrition.

[25] Summer S.S., Brehm B.J., Benoit S.C., D’Alessio D.A. (2011). Adiponectin changes in relation to the macronutrient composition of a weight-loss diet. Obes. Silver Spring.

[26] Rankin J.W., Turpyn A.D. (2007). Low carbohydrate, high fat diet increases C-reactive protein during weight loss. J. Am. Coll. Nutr..

[27] Ruth M.R., Port A.M., Shah M., Bourland A.C., Istfan N.W., Nelson K.P., Gokce N., Apovian C.M. (2013). Consuming a hypocaloric high fat low carbohydrate diet for 12 weeks lowers C-reactive protein, and raises serum adiponectin and high density lipoprotein-cholesterol in obese subjects. Metabolism.

[28] Sharman M.J., Volek J.S. (2004). Weight loss leads to reductions in inflammatory biomarkers after a very-low-carbohydrate diet and a low-fat diet in overweight men. Clin. Sci. Lond..

[29] Forsythe C.E., Phinney S.D., Fernandez M.L., Quann E.E., Wood R.J., Bibus D.M., Kraemer W.J., Feinman R.D., Volek J.S. (2008). Comparison of low fat and low carbohydrate diets on circulating fatty acid composition and markers of inflammation. Lipids.

[30] Cardillo S., Seshadri P., Iqbal N. (2006). The effects of a low-carbohydrate versus low-fat diet on adipocytokines in severely obese adults: Three-year follow-up of a randomized trial. Eur. Rev. Med. Pharmacol. Sci..

[31] Seshadri P., Samaha F.F., Stern L., Ahima R.S., Daily D., Iqbal N. (2005). Adipocytokine changes caused by low-carbohydrate compared to conventional diets in obesity. Metab. Syndr. Relat. Disord..

[32] Wycherley T.P., Brinkworth G.D., Keogh J.B., Noakes M., Buckley J.D., Clifton P.M. (2010). Long-term effects of weight loss with a very low carbohydrate and low fat diet on vascular function in overweight and obese patients. J. Intern. Med..

[33] National Cholesterol Education Program (NCEP) (2002). Expert panel on detection, evaluation, and treatment of high blood cholesterol in adults (Adult Treatment Panel III). Third report of the NCEP expert panel on detection, evaluation, and treatment of high blood cholesterol in adults (Adult Treatment Panel III) final report. Circulation.

[34] American Heart Association (AHA) (2000). AHA dietary guidelines revision 2000: A statement for healthcare professionals from the Nutrition Committee of the AHA. Circulation.

[35] Nutrition Data System for Research (2005). The Minnesota Nutrition Data System.

[36] Larosa J.C., Fry A.G., Muesing R., Rosing D.R. (1980). Effects of high-protein, low-carbohydrate dieting on plasma lipoproteins and body weight. J. Am. Diet. Assoc..

[37] Molenberghs G., Kenward M. (2007). Missing Data in Clinical Studies.

[38] Lange T., Vansteelandt S., Bekaert M. (2012). A simple unified approach for estimating natural direct and indirect effects. Am. J. Epidemiol..

[39] Cole S.R., Hernan M.A. (2002). Fallibility in estimating direct effects. Int. J. Epidemiol..

[40] Baron R.M., Kenny D.A. (1986). The moderator-mediator variable distinction in social psychological research: Conceptual, strategic, and statistical considerations. J. Personal. Soc. Psychol..

[41] Bessesen D.H. (2001). The role of carbohydrates in insulin resistance. J. Nutr..

[42] Ziemke F., Mantzoros C.S. (2010). Adiponectin in insulin resistance: Lessons from translational research. Am. J. Clin. Nutr..

[43] Ryan A.S., Nicklas B.J., Berman D.M., Elahi D. (2003). Adiponectin levels do not change with moderate dietary induced weight loss and exercise in obese postmenopausal women. Int. J. Obes. Relat. Metab. Disord..

[44] Keogh J.B., Brinkworth G.D., Clifton P.M. (2007). Effects of weight loss on a low-carbohydrate diet on flow-mediated dilatation, adhesion molecules and adiponectin. Br. J. Nutr..

[45] Kelly K.R., Navaneethan S.D., Solomon T.P., Haus J.M., Cook M., Barkoukis H., Kirwan J.P. (2014). Lifestyle-induced decrease in fat mass improves adiponectin secretion in obese adults. Med. Sci. Sports Exerc..

[46] Gavrila A., Chan J.L., Yiannakouris N., Kontogianni M., Miller L.C., Orlova C., Mantzoros C.S. (2003). Serum adiponectin levels are inversely associated with overall and central fat distribution but are not directly regulated by acute fasting or leptin administration in humans: Cross-sectional and interventional studies. J. Clin. Endocrinol. Metab..

[47] Chen J.H., Ouyang C., Ding Q., Song J., Cao W., Mao L. (2015). A moderate low-carbohydrate low-calorie diet improves lipid profile, insulin sensitivity and adiponectin expression in rats. Nutrients.

[48] Kim J.A., Montagnani M., Koh K.K., Quon M.J. (2006). Reciprocal relationships between insulin resistance and endothelial fysfunction: Molecular and pathophysiological mechanisms. Circulation.

[49] Calabresi L., Gomaraschi M., Villa B., Omoboni L., Dmitrieff C., Franceschini G. (2002). Elevated soluble cellular adhesion mlecules in subjects with low HDL-cholesterol. Arterioscler. Thromb. Vasc. Biol..

[50] Glushakova O., Kosugi T., Roncal C., Mu W., Heinig M., Cirillo P., Sanchez-Lozada L.G., Johnson R.J., Nakagawa T. (2008). Fructose induces the inflammatory molecule ICAM-1 in endothelial cells. J. Am. Soc. Nephrol..

